# Patient experiences of opposite gender nurse–patient interactions at public health facilities in Namibia

**DOI:** 10.4102/curationis.v49i1.2824

**Published:** 2026-04-14

**Authors:** Nestor Tomas, Secilia Kayangura

**Affiliations:** 1 School of Nursing and Public Health, Faculty of Health Sciences and Veterinary Medicine, University of Namibia, Rundu, Namibia

**Keywords:** disclosure, health facilities, Namibia, patient comfort, personal satisfaction, privacy, religion, trust

## Abstract

**Background:**

The imbalance in nurse-to-patient ratio and shortage of nurses is the main drive for care provision by a nurse of the opposite gender. On the other hand, while nurses must recognise and respect the role of patients as partners, the nurse–patient relationship with the opposite gender in Namibia is limited because of the cultural and religious beliefs.

**Objectives:**

To explore and describe patients’ experiences on opposite gender nurse–patient interactions at selected public health facilities in Namibia.

**Method:**

An exploratory descriptive qualitative design was employed to collect data from 14 purposively recruited patients who received care from nurses of opposite gender between June 2022 and September 2022. The interviews were audio-recorded, transcribed, and analysed using inductive thematic analysis. The study adhered to the Consolidated Criteria for Reporting Qualitative Research reporting guideline.

**Results:**

Two major themes emerged: (1) barriers to opposite-gender nurse–patient interactions, which include culturally insensitive care, gender-related privacy concerns, and age-insensitive care; and (2) strategies to improve nurse–patient interactions, describing the strategies to promote privacy and patient comfort, create a safe space for disclosure and implement gender-concordant care to address the identified barriers.

**Conclusion:**

The findings of this study revealed that patients had negative experiences with opposite gender nurse–patient interactions. The study recommends the provision of privacy, creating a safe space for disclosure and providing gender-concordant care to create trust and improved satisfaction with nursing care.

**Contribution:**

The study provides essential insights into the cultural nuances of opposite gender nurse–patient interactions within the public health sector.

## Introduction

Patient-nurse interaction is an essential issue in all areas of nursing practice, including prevention, treatment, rehabilitation, education and health promotion (Vatandost et al. 2020a). Nurse–patient interactions encapsulate the professional relationship designed to help nurses assess, plan and provide care that addresses the patient’s fundamental needs (Vujanić et al. 2022). Because of the shortage of nurses and the high nurse-to-patient ratio, it is necessary for both males and females to join the profession (Sharifi et al. 2021). However, gender differences have been noted as a barrier to the patient-nurse relationship (Vatandost et al., 2020b). Many prior studies focused predominantly on general nurse–patient communication (Azarabadi et al. 2024; Babaii, Mohammadi & Sadooghiasl 2021; Kwame & Petrucka 2021), patients’ attitudes towards cross-gender interactions (Sharifi et al. 2021), and male nurses’ challenges in caring for the opposite gender (Abdulla, Naqi & Jassim 2022; Vatandost et al. 2020b), rather than focusing on patients’ experiences of opposite-gender nurse–patient interactions.

Globally, nursing has been acknowledged as a predominantly female profession (Gauci et al. 2023; Valiee, Moradi & Vatandost 2022). The existing imbalance in the nurse-to-patient ratio has necessitated the recruitment of additional nurses, including male nurses (Gauci et al. 2023; Sharifi et al. 2021). Nonetheless, there is an expectation for both male and female nurses to recognise and respect the role of patients as partners in their care (Asante, Korsah & Amoako 2023; Sharifi et al. 2021; Valiee et al. 2022). While nurses can offer patients the emotional and psychological support essential for their healing process, the gender of the nurse may impact the patient’s experiences (Asante et al. 2023). Various factors, including age, language, cultural norms, interpersonal skills, patients’ preferences and religion, shape individual perceptions and experiences (Alsadaan et al. 2021; Asante et al. 2023; Sharifi et al. 2021; Soine 2019; Valiee et al. 2022). Without the patient’s acceptance of the nurse’s care, adherence to the treatment regimen and satisfaction with the nursing care provided become unattainable (Sharifi et al. 2021).

The prevailing assumption is that patients of any gender prefer care from female nurses; however, some argue that female nurses encounter challenges similar to those faced by their male counterparts (Asante et al. 2023; Vatandost et al. 2020b). For instance, research indicates that male patients may refuse to allow a female nurse to administer an enema (Asante et al. 2023). Conversely, male nurses also raise concerns about providing care to female patients when such care necessitates physical contact (Cheng et al. 2018). Additionally, male nurses often face challenges such as discrimination and the refusal of care by patients of the opposite sex (Cheng et al. 2018; Gao et al. 2019; Valiee et al. 2022; Wong et al. 2025; Younas et al. 2022). Furthermore, evidence suggests that male nurses not only possess essential leadership skills, but are also particularly adept at managing aggressive and irritable patients (Mao et al. 2021; Prosen 2022).

Currently, the nursing profession includes both men and women; however, gender preferences have been reported as a barrier to patient-nurse interactions (Bakhshi et al. 2024; Sharifi et al. 2021). There is a prevailing perception that male nurses possess different attributes compared to their female counterparts, particularly in areas such as leadership qualities, emotional sensitivity, technical ability and the influence of their sexuality (Aca et al. 2025; Mao et al. 2021; Martínez-Morato et al. 2021; Prosen 2022).

However, the power dynamics and vulnerabilities inherent in the patient-provider relationship place patients in precarious situations during physical examinations and nursing or medical procedures (Okonji et al. 2024). The perpetration of sexual abuse by healthcare professionals not only constitutes a profound breach of trust and professional ethics but also undermines the foundational principles of medical ethics, namely respect for patient autonomy, beneficence and non-maleficence (Cheraghi et al. 2023; Okonji et al. 2024). Reports indicate documented cases of sexual assault, specifically rape, perpetrated by opposite-gender nurses against female patients within Namibia (Links 2019). Understanding patients’ experiences will aid policymakers, hospital management, and training institutions in developing strategies to promote interactions between opposite-gender nurses and patients, ultimately leading to improved patient safety and satisfaction. This study described patients’ experiences with opposite gender nurse–patient interaction at selected public health facilities in Namibia.

## Research methods and design

### Design and setting

A qualitative exploratory descriptive design was employed to explore and describe patients’ experiences with opposite-gender nurse interactions at the selected public health facilities. Drawing on previous studies (Asante et al., 2023; Asmaningrum et al., 2020; Azarabadi et al., 2024), this design was considered suitable because it allowed for a thorough and in-depth exploration of the students’ experiences with opposite gender nurse–patient interaction at selected public health facilities in Namibia. The settings were one intermediate hospital and two clinics in Namibia’s Kavango East region. These facilities, located in Rundu, are accredited training institutions for nurses and midwives and employ approximately 300 healthcare professionals. The town’s strategic location along a major highway and its proximity to several neighbouring countries make it one of Namibia’s fastest-growing towns.

### Population and sampling

A purposive sampling method was used to recruit 10 participants at the selected public health facility. Interviewing a sample size of 5–24 participants has been documented as a suitable sample size for qualitative studies, leading to thematic and code saturation (Hennink & Kaiser 2022). To be eligible for this study, participants had to be: (1) male or female patients; (2) aged 18 and above who visited the selected public health facilities; and (3) who interacted with nurses of the opposite gender. The study excluded two participants who were mentally unfit.

### Data collection

Data collection was conducted in June 2022 using a semi-structured interview guide to allow the patients to share their experiences regarding opposite gender nurse–patient interactions. The principal researcher (Secilia Kayangura & Nestor Tomas) opted for this particular period in order to gather information before the conclusion of the academic year. A semi-structured interview guide for face-to-face was developed in accordance with the study’s objectives and in line with extant literature (Sharifi et al. 2021; Vatandost et al. 2020b). The interview allowed participants sufficient opportunity to develop and share their affective and cognitive accounts of their experiences (Adeoye-Olatunde & Olenik 2021; Karatsareas 2022) on opposite gender nurse–patient interactions.

One of the authors (Secilia Kayangura) requested the in-charge of the facility for a room where the individual face-to-face interviews took place. The author then approached potential participants at the hospital, explained the purpose and significance of the study, and requested their voluntary participation and consent. Participants were informed that the interviews would be recorded using an audio recording.

Two key questions were posed: (1) What are the patients’ experiences of opposite gender nurse–patient interactions? (2) What suggestions do you have for improving opposite gender nurse–patient interactions at the selected health facility? Probing questions such as ‘Could you clarify?’, ‘What were your positive and negative experiences?’ and ‘Could you provide more detail?’ were utilised to obtain more comprehensive information from the participants.

Ten interviews were conducted, after which no new themes or codes emerged, indicating thematic saturation. This aligns with research by Hennink and Kaiser (2022), which suggests that saturation is typically reached around the eighth interview. Each interview, which lasted between 30 min and 35 min, was assigned a code from P1 to P10 to identify the recordings and field notes.

### Data analysis

The data were transcribed verbatim by the second author (Secilia Kayangura). To identify patterns in the data, the six-step inductive thematic analysis method outlined by Braun and Clarke (2023) was employed. To familiarise with the data, the second author (Secilia Kayangura) thoroughly listened to the recordings repeatedly, and examined transcripts before it was entered into a Microsoft Word document. To generate initial codes, relevant pieces of data from various sources were read and analysed to make sense of them, including documenting any ideas that came to the researchers’ minds. To facilitate analysis, the second author reviewed the transcripts and highlighted different segments in various colours. To generate initial themes, different codes were then put together in a table. Subsequently, a cross-verification of the codes was done prior to their integration of observational/field notes. Similar codes were clustered together and labelled into meaningful groups of subthemes before reviewing them by going through the entire coded data set and assigning them to the corresponding segments of text. The data organisation was assessed to identify any new themes. To define and name the themes, the relationships between subthemes and themes were recognised to reduce the overall list. Grouping them together and indicating their relationships helped establish the connections.

### Data quality

The study utilised the following quality criteria for trustworthiness: credibility, dependability, conformability and transferability (Lincoln & Guba 1985; Pratt, Sonenshein & Feldman 2022). Credibility was established through purposive sampling methods, ensuring that participants met the inclusive criteria, and by recording the interviews to prevent data loss. Although no discrepancies were identified, member checking was conducted to confirm the accuracy and truthfulness of the findings. Consequently, the initial findings were upheld without modification. For credibility, the research team, comprising a novice researcher (Secilia Kayangura) and a senior researcher (Nestor Tomas) with experience in qualitative research and a record of publishing qualitative studies. Dependability was ensured through the use of recorded data and an inquiry audit trail, which was performed by the research supervisor to verify the accuracy of the findings. The study ensured transferability by providing detailed descriptions of the procedures used for data collection (Braun & Clarke 2021). The data reported in this study were based on the study’s objectives and are representative of the participants’ experiences of opposite-gender nurse–patient interactions at the selected health facility in Namibia. To prevent premature data analysis, the researchers allocated sufficient time to thoroughly examine the data before conducting the analysis in manageable segments. This approach facilitated a comprehensive understanding of the contexts of the participants’ experiences, thereby enhancing coding decisions and avoiding the creation of superficial codes (Braun & Clarke 2021; Inayat et al. 2024). Reflexivity was practised by documenting the researchers’ assumptions to prevent them from influencing the participants’ responses.

### Ethical considerations

Ethical clearance to conduct this study was obtained from the University of Namibia’s School of Nursing and Public Health Ethical Committee on 13 June 2022. The ethical approval number is SoN 72/2022. Before participating, all individuals gave their written informed consent and were informed of their right to withdraw from the study at any time if they so wished. The research was conducted in line with the revised Declaration of Helsinki, which provides ethical guidelines for medical research involving human subjects.

## Results

### Participants’ demographics

Out of 14 participants, 64% (*n* = 9) were females, while 36% (*n* = 5) were males. The mean age of the participants was 36 years. The age distribution showed that the majority, 57% (*n* = 8), were between 22 and 35 years old, followed by 42% (*n* = 6) in the 41- to 56-year age bracket. Regarding educational background, 57% (*n* = 8) had completed junior or senior certificates, while 43% (*n* = 6) held a diploma or degree. All participants (100%, *n* = 14) self-identified as being of the black race (Table 1).

**TABLE 1:** Participants demographics characteristics.

Participant no	Gender	Age (years)	Educational level	Race
P1	Male	29	Degree/diploma	Black person
P2	Female	24	Junior/senior certificates	Black person
P3	Female	22	Junior/senior certificates	Black person
P4	Female	53	Junior/senior certificates	Black person
P5	Female	27	Degree/diploma	Black person
P6	Male	41	Junior/senior certificates	Black person
P7	Female	23	Degree/diploma	Black person
P8	Female	28	Junior/senior certificates	Black person
P9	Male	26	Degree/diploma	Black person
P10	Female	56	Junior/senior certificates	Black person
P11	Female	45	Degree/diploma	Black person
P12	Male	35	Junior/senior certificates	Black person
P13	Female	30	Degree/diploma	Black person
P14	Female	50	Junior/senior certificates	Black person

### Themes and subthemes

The study identified two major themes (Table 2). The first theme on barriers to opposite gender nurse–patient interactions encompasses culturally insensitive care, gender-related privacy concerns and age-insensitive care. The second theme focused on the strategies to improve opposite gender nurse–patient interaction detailing promoting privacy and patient comfort, creating a safe space for disclosure and implementing gender-concordant care as measures to address barriers to opposite gender nurse–patient interactions.

**TABLE 2:** Themes and subthemes.

Themes	Subthemes
1. Barriers to opposite gender nurse–patient interactions	1.1. Cultural insensitive care 1.2. Gender related privacy concerns 1.3. Age insensitive care
2. Strategies to improve opposite gender nurse–patient interactions	2.1. Promoting privacy and patient comfort 2.2. Implementation of gender-concordant care

#### Theme 1: Barriers to opposite gender nurse–patient interactions

This theme emerged when participants were asked to share their experiences regarding opposite gender nurse–patient interactions. All participants reported having negative experiences with opposite gender nurse–patient interactions. These negative experiences were grouped into three key subthemes: culturally insensitive care, privacy concerns and age-insensitive care.

**Subtheme 1.1: Cultural insensitive care:** One-reason participants were affected by opposite gender interaction was because of their cultural beliefs. Their cultural values and norms restrict them from being examined by nurses of the opposite gender, especially during a physical examination:

‘Based on my cultural beliefs, a female is not allowed to see the private parts of a male to whom she is not married; this is considered an abomination or a taboo in our culture.’ (P1, male, 29 years)

‘[*Frowning*], In my culture, it’s disrespectful. A female is not allowed to show her body to just anyone. Although I was in pain, I felt really uncomfortable, and it was an insult to me to have two male nurses assisting me during my delivery.’ (P5, male, 27 years)

**Subtheme 1.2: Gender-related privacy concerns:** When discussing their experiences, participants expressed concerns about privacy violations if they were required to undergo a physical examination by nurses of the opposite gender. The presence of multiple nurses, particularly of the opposite gender, in a room can be intimidating, overriding patients’ rights to privacy and dignity and preventing open communication:

‘Oh, I can start by mentioning a lack of privacy. For instance, when you enter a room with several nurses of the opposite gender, I felt uncomfortable being examined. I was obviously ashamed to share my problems with someone of the opposite gender.’ (P1, 29 years, male)

‘To be honest, when I was at the hospital, there were many female nurses, and I found it tough to share my problems with them. Instead of telling them the real issue, I ended up lying.’ (P6, 41 years, male)

**Subtheme 1.3: Age-insensitive care:** Age-insensitive care was a recurring subtheme, with participants describing discomfort and misunderstandings when receiving care from nurses who were significantly younger than them and of the opposite gender:

‘[*Grimacing*], I think age is very important. At my age, if I walk into a consulting room and find a male nurse who is much older than me, I will not be very open about sharing my situation, especially on sexual health.’ (P2, 24 years, female)

‘Upon entering the room, you will discover that you are older than the nurse. I believe that even if I explain my problem to the boy I see here, he will not be able to communicate effectively, as I doubt he will understand my issue.’ (P4, 53 years, female)

One participant narrated how sharing a problem with a nurse who was younger than him was uncomfortable and shameful:

‘When the nurse is younger than the patient, it can lead to a lack of trust in sharing and allowing that nurse to see or touch my body, as it feels disrespectful. I may even end up undermining the nurse. I feel like I am showing my grown-up son my nakedness during the examination.’ (P10, 56 years, female)

#### Theme 2: Strategies to improve opposite gender nurse–patient interactions

Participants suggested several strategies to improve interactions between nurses and patients of the opposite gender. Data analysis revealed three main subthemes: promoting privacy and comfort, creating a safe space for disclosure and implementing gender-concordant care.

**Subtheme 2.1: Promoting privacy and patient comfort:** This subtheme captures the core issue of patients’ feelings of discomfort as a result of a lack of privacy. Participants recommended maintaining a minimal number of nurses of the opposite gender in the consultation rooms to ensure privacy during interactions and to uphold patients’ dignity:

‘Privacy must be considered. It is important to ensure that if a nurse of the opposite gender is the only option, then only one nurse should be in that room to attend to the patient, not several.’ (P7, 23 years, female)

‘If there are two nurses of the opposite gender to the patient, then at least one should leave the room.’ (P8, 28 years, female)

Other participants emphasise the need for nurses to actively create a space where patients feel safe to share their concerns. The actions of ‘excusing’ themselves and ‘respecting cultural beliefs’ are deliberate strategies to achieve this:

‘In situations like entering a room with more than two nurses present, the necessary improvement is that those two nurses must excuse themselves. There should be at least one nurse available to ensure that a patient feels comfortable and free to communicate, allowing them to express their concerns about why they are there.’ (P14, 50 years, female)

‘They need to respect that some cultures prevent the opposite gender from seeing, touching, or asking older people certain things. This consideration is important to allow patients to open up to those they feel are fit to listen and address their health issues.’ (P11, 35 years, female)

**Subtheme 2.2: Implementation of gender-concordant care:** This subtheme focuses on the importance of gender matching between the nurse and the patient. Participants suggested that when a patient is cared for by a nurse of the same gender, it can improve comfort and communication. This approach is believed to create a more trusting environment, where patients feel more at ease discussing sensitive issues, ultimately leading to higher satisfaction with their nursing care:

‘I suggest there should be an option for patients to choose who treats them. Patients should be asked if they prefer to be treated by someone of the same gender or the opposite gender.’ (P13, 30 years, female)

‘Actually, I will say that male patients need to be cared for by someone of the same gender, as this will allow them to share the problems they came for.’ (P12, 35 years, male)

‘I think it’s also important to consider being examined by someone of the same gender. If I’m a teenager and the person treating me is of the opposite gender, it is unimaginable. Having someone of the same gender creates a more comfortable atmosphere between the patient and the nurse.’ (P3, 22 years, female)

## Discussion

The recent study explored and described patients’ experiences on opposite gender nurse–patient interactions at selected public health facilities in Namibia. The study identified various barriers to opposite gender nurse–patient interactions. A key finding from the study revealed that receiving care from a nurse of the opposite gender was insensitive to the participants’ cultural values and norms, which prohibit individuals from seeing the naked bodies of persons who are not their spouses; thus, it was labelled as a taboo. This finding conflicts with the Claeys et al. (2021) study, which claims that nurses must provide culturally sensitive care to develop a positive and healing relationship with patients from diverse ethnic and cultural backgrounds. To provide culturally sensitive care, nurses must navigate the various social, economic, and historical factors that influence global nursing practices (Mohd et al. 2024).

This requires nurses to follow global health systems while respecting cultural attitudes towards healthcare and understanding their own roles. O’Brien et al. (2021) argue that providing high-quality healthcare is impossible without taking considering a patient’s culture, values and personal health beliefs. Therefore, it is crucial for both healthcare institutions and providers to respect the diversity that exists within different cultural groups.

Another barrier to opposite gender nurse–patient interactions in the current study was age-insensitivity care. Participants expressed concerns of gender related privacy violations during physical examination, discomfort and misunderstandings by younger nurses of the opposite gender. These findings agree with Sharifi et al. (2021) study, which postulates negative attitudes from both male and female patients regarding receiving care from nurses of the opposite gender. While older adults continue to be the primary consumers of healthcare services in Namibia (Tomas, Ashipala & Tomas 2021), they exhibit similar demographic and sociocultural characteristics to many other elderly individuals across African nations (Fuseini et al. 2023). Evidence indicates that it is considered taboo for individuals of the opposite gender to view another person naked unless they are married (Fuseini et al. 2023; Rangolo, Tshitangano & Olaniyi 2021). When older patients are cared for by nurses of the opposite gender, they often feel vulnerable, which can lead to feelings of shyness or shame, especially during intimate tasks like physical exams (Tohit & Haque 2024; Xiarchi et al. 2024). While some research (Xiarchi et al. 2024) suggests that older male patients may prefer female nurses, our study found that both male and female patients were uncomfortable being cared for by a nurse of the opposite gender. This discomfort was particularly strong when it came to physical examinations or discussing sexual health issues with younger nurses. Moreover, according to a recent study by Shakwane (2022), elderly patients disapprove of receiving intimate care, such as bathing, from younger nurses because of the age gap. The inability to openly discuss sexual health with younger providers could be attributed to deep-rooted cultural beliefs and religious beliefs (Hinchliff et al. 2021; Tohit & Haque 2024).

As a result of the feelings of privacy violation, a recent study found that patients had negative experiences with opposite gender interactions during physical examinations. Patients reported discomfort with exposing their bodies to nurses of the opposite gender. This lack of respect for patient privacy contradicts findings from other studies (Asmaningrum, Kurniawati & Tsai 2020; Öztürk et al. 2021). The literature suggests that this disregard for privacy could be caused by attitudes towards gossiping, a lack of information, or inadequate training about patient privacy and rights (Ceylan & Çetinkaya 2020). In agreement, recent studies (Sharifi et al. 2021; Yilmaz & Celik 2022) identified several recurring issues in healthcare communication, including a lack of two-way dialogue, nurses’ disregard for patient privacy, and biased behaviour towards patients. Privacy contributes to the preservation of a sense of reverence and dignity; therefore, it is essential for establishing and maintaining a respectful and effective clinical relationship and is a crucial component of patient-centred care (Valizadeh & Ghasemi 2020; Zhang et al. 2021). Moreover, observing a patient’s privacy helps them feel safe, which in turn strengthens their trust in the nurse (Feng et al. 2024; Moosavi et al. 2023). Non-compliance with privacy issues may exacerbate stress, open communication and hinder patients’ cooperation with medical staff (Saleem et al. 2022). To gain a comprehensive overview of patients’ privacy, further studies should investigate nurses’ knowledge of patients’ privacy and patients’ rights in Namibia.

To improve patient comfort, communication, and dignity, the study suggests that healthcare facilities should minimise the number of opposite gender nurses in consultation rooms and, whenever possible, have patients cared for by a same-gender nurse. This highlights the critical need for nurses to actively foster a safe environment where patients feel comfortable sharing their concerns. Numerous studies have consistently demonstrated a strong preference amongst patients for same-gender nurse–patient interactions (Asante et al. 2023; Cheng et al. 2018; Lee et al. 2024; Prosen 2022; Wong et al. 2025; Younas et al. 2022). This preference often stems from concerns about privacy, as a gender difference between a nurse and a patient can compromise a patient’s sense of privacy (Vatandost et al. 2020b). This is particularly true for female patients, who tend to be more sensitive to physical touch and feel more comfortable with healthcare providers of the same gender (Alhomayani et al. 2025; Fink et al. 2020). This heightened anxiety amongst female patients may be linked to a fear of sexual assault, given media reports of rape cases perpetrated by healthcare providers (Okonji et al. 2024).

This study elucidated significant impediments to opposite gender nurse–patient interactions and delineated strategies for integrating patient preferences into the planning of nursing care delivery within public health facilities in Namibia.

### Strengths and limitations

The study’s primary strength is its qualitative design, which allowed for a deep exploration of patients’ personal experiences. By using in-depth interviews, the researchers were able to gather detailed information on sensitive subjects concerning cultural beliefs and privacy. The use of a purposive sampling method ensured that all participants had firsthand experience with opposite-gender nurse–patient interactions, directly addressing the study’s objectives. However, the study had a few limitations. A key limitation of the study is its focus on a single, ‘selected’ public health facility in an urban setting, limiting the transferability of the results to other healthcare settings.

## Conclusion

This study’s findings indicate that patients’ negative experiences of opposite gender nurse–patient interactions were driven by culturally insensitive care, concerns about privacy and age-related discomfort. To overcome these challenges, patients suggested implementing strategies such as promoting privacy and comfort by limiting the number of nurses in the room, creating a safe space for disclosure that respects cultural beliefs, and providing gender-concordant care where patients can choose a nurse of the same gender. The study recommends that implementing strategies suggested by patients can lead to better communication, increased trust, and improved satisfaction with nursing care. Further studies should focus on large cross-sectional research to investigate nurses’ knowledge of privacy and patients’ rights in Namibia.

## References

[CIT0001] Abdulla, N.M., Naqi, R.J. & Jassim, G.A., 2022, ‘Barriers to nurse-patient communication in primary healthcare centers in Bahrain: Patient perspective’, *International Journal of Nursing Sciences* 9(2), 230–235. 10.1016/j.ijnss.2022.03.006 35509693 PMC9052254

[CIT0002] Aca, Z., Kırcal-Şahin, A., Özdemir, A. & Kaymakcı, Y.S., 2025, ‘Gender stereotypes and professional experiences of female nurses in Türkiye’, *Frontiers in Public Health* 13, 1538517. 10.3389/fpubh.2025.1538517 39925754 PMC11803633

[CIT0003] Adeoye-Olatunde, O.A. & Olenik, N.L., 2021, ‘Research and scholarly methods: Semi-structured interviews’, *Journal of the American College of Clinical Pharmacy* 4(10), 1358–1367. 10.1002/jac5.1441

[CIT0004] Alhomayani, K.M., Bukhary, H.A., Aljuaid, F.I., Alotaibi, T.A., Alqurashi, F.S., Althobaiti, K.N. et al., 2025, ‘Gender preferences in healthcare: A study of Saudi patients’ physician preferences’, *Patient Preference and Adherence* 2025, 295–303. 10.2147/PPA.S494766 PMC11806673 39926247

[CIT0005] Alsadaan, N., Jones, L.K., Kimpton, A. & DaCosta, C., 2021, ‘Challenges facing the nursing profession in Saudi Arabia: An integrative review’, *Nursing Reports* 11(2), 395–403. 10.3390/nursrep11020038 34968216 PMC8608082

[CIT0006] Asante, A.O., Korsah, K.A. & Amoako, C., 2023, ‘Does the gender of nurses matter to patients? A qualitative analysis of gender preferences of patients’, *SAGE Open Medicine* 11, 20503121231182514. 10.1177/205031212311825 37456084 PMC10338727

[CIT0007] Asmaningrum, N., Kurniawati, D. & Tsai, Y.F., 2020, ‘Threats to patient dignity in clinical care settings: A qualitative comparison of Indonesian nurses and patients’, *Journal of Clinical Nursing* 29(5–6), 899–908. 10.1111/jocn.15144 31855306

[CIT0008] Azarabadi, A., Bagheriyeh, F., Moradi, Y. & Orujlu, S., 2024, ‘Nurse-patient communication experiences from the perspective of Iranian cancer patients in an outpatient oncology clinic: A qualitative study’, *BMC Nursing* 23(1), 682. 10.1186/s12912-024-02339-4 39334158 PMC11438122

[CIT0009] Babaii, A., Mohammadi, E. & Sadooghiasl, A., 2021, ‘The meaning of the empathetic nurse–patient communication: A qualitative study’, *Journal of Patient Experience* 8, 23743735211056432. 10.1177/23743735211056432 34869836 PMC8640307

[CIT0010] Bakhshi, H., Shariati, M.J., Basirinezhad, M.H. & Ebrahimi, H., 2024, ‘Comparison of barriers to effective nurse-patient communication in COVID-19 and non-COVID-19 wards’, *BMC Nursing* 23(1), 328. 10.1186/s12912-024-01947-4 38755576 PMC11097547

[CIT0011] Braun, V. & Clarke, V., 2021, ‘To saturate or not to saturate? Questioning data saturation as a useful concept for thematic analysis and sample-size rationales’, *Qualitative Research in Sport, Exercise and Health* 13(2), 201–216. 10.1080/2159676X.2019.1704846

[CIT0012] Braun, V. & Clarke, V., 2023, ‘Toward good practice in thematic analysis: Avoiding common problems and be(com)ing a knowing researcher’, *International Journal of Transgender Health* 24(1), 1–6. 10.1080/26895269.2022.2129597 36713144 PMC9879167

[CIT0013] Ceylan, S.S. & Çetinkaya, B., 2020, ‘Attitudes towards gossip and patient privacy among paediatric nurses’, *Nursing Ethics* 27(1), 289–300. 10.1177/0969733019845124 31088205

[CIT0014] Cheng, M.L., Tseng, Y.H., Hodges, E. & Chou, F.H., 2018, ‘Lived experiences of novice male nurses in Taiwan’, *Journal of Transcultural Nursing* 29(1), 46–53. 10.1177/1043659616676318 27815552

[CIT0015] Cheraghi, R., Valizadeh, L., Zamanzadeh, V., Hassankhani, H. & Jafarzadeh, A., 2023, ‘Clarification of ethical principle of the beneficence in nursing care: An integrative review’, *BMC Nursing* 22(1), 89. 10.1186/s12912-023-01246-4 36997958 PMC10061877

[CIT0016] Claeys, A., Berdai-Chaouni, S., Tricas-Sauras, S. & De Donder, L., 2021, ‘Culturally sensitive care: Definitions, perceptions, and practices of health care professionals’, *Journal of Transcultural Nursing* 32(5), 484–492. 10.1177/1043659620970625 33150857

[CIT0017] Feng, Y., Liu, C., Tao, S., Wang, C., Zhang, H., Liu, X. et al., 2024, ‘Developing and validating the nurse-patient relationship scale (NPRS) in China’, *BMC Nursing* 23(1), 255. 10.1186/s12912-024-01941-w 38649929 PMC11034141

[CIT0018] Fink, M., Klein, K., Sayers, K., Valentino, J., Leonardi, C., Bronstone, A. et al., 2020, ‘Objective data reveals gender preferences for patients’ primary care physician’, *Journal of Primary Care & Community Health* 11, 2150132720967221. 10.1177/2150132720967221 PMC7786418 33111633

[CIT0019] Fuseini, A.G., Rawson, H., Ley, L. & Kerr, D., 2023, ‘Patient dignity and dignified care: A qualitative description of hospitalised older adults perspectives’, *Journal of Clinical Nursing* 32(7–8), 1286–1302. 10.1111/jocn.16286 35322497

[CIT0020] Gao, Y., Cheng, S., Madani, C. & Zhang, G., 2019, ‘Educational experience of male students in a baccalaureate nursing program in China’, *Nurse Education in Practice* 35, 124–129. 10.1016/j.nepr.2019.02.006 30785064

[CIT0021] Gauci, P., Luck, L., O’Reilly, K. & Peters, K., 2023, ‘Workplace gender discrimination in the nursing workforce – An integrative review’, *Journal of Clinical Nursing* 32(17–18), 5693–5711. 10.1111/jocn.16684 36922724

[CIT0022] Hennink, M. & Kaiser, B.N., 2022, ‘Sample sizes for saturation in qualitative research: A systematic review of empirical tests’, *Social Science & Medicine* 292(2022), 114523. 10.1016/j.socscimed.2021.114523 34785096

[CIT0023] Hinchliff, S., Fileborn, B., Alba, B., Lyons, A., Minichiello, V., Barrett, C. et al., 2021, ‘Talking about sex with friends: Perspectives of older adults from the sex, age & me study in Australia’, *Culture, Health & Sexuality* 23(3), 367–382. 10.1111/hex.12418 32609066

[CIT0024] Inayat, S., Younas, A., Fàbregues, S. & Ali, P., 2024, ‘Premature closure of analysis in qualitative research: Identifying features and mitigation strategies’, *International Journal of Qualitative Methods* 23, 1–10. 10.1177/16094069241234187

[CIT0025] Karatsareas, P., 2022, ‘Semi-structured interviews’, in R. Kircher & L. Zipp (eds.), *Research methods in language attitudes*, pp. 99–113, Cambridge University Press, Cambridge, UK.

[CIT0026] Kwame, A. & Petrucka, P.M., 2021, ‘A literature-based study of patient-centered care and communication in nurse-patient interactions: Barriers, facilitators, and the way forward’, *BMC Nursing* 20(1), 158. 10.1186/s12912-021-00684-2 34479560 PMC8414690

[CIT0027] Lee, C.Y., Lee, C.H., Lai, H.Y. & Yau, S.Y., 2024, ‘An investigation of patient preferences and gender dynamics of neurosurgeon selection in Taiwan: A mixed-method study’, *World Neurosurgery* 186, 43–49. 10.1016/j.wneu.2024.03.068 38514029

[CIT0028] Lincoln, Y.S. & Guba, E.G., 1985, *Naturalistic inquiry*, Sage, Newbury Park, CA.

[CIT0029] Links, A., 2019, ‘Safety not guaranteed: Raped in hospital’, *Sister Namibia* 31(1), 12–14, viewed 20 May 2022, from https://go.gale.com/ps/i.do?id=GALE%7CA588991641&sid=googleScholar&v=2.1&it=r&linkaccess=abs&issn=10269126&p=AONE&sw=w&userGroupName=anon%7Ec69a2b32&aty=open-web-entry.

[CIT0030] Mao, A., Cheong, P.L., Van, I.K. & Tam, H.L., 2021, ‘“I am called girl, but that doesn’t matter” – Perspectives of male nurses regarding gender-related advantages and disadvantages in professional development’, *BMC Nursing* 20(1), 24. 10.1186/s12912-021-00539-w 33468102 PMC7815446

[CIT0031] Martínez-Morato, S., Feijoo-Cid, M., Galbany-Estragués, P., Fernández-Cano, M.I. & Arreciado Marañón, A., 2021, ‘Emotion management and stereotypes about emotions among male nurses: A qualitative study’, *BMC Nursing* 20(1), 114. 10.1186/s12912-021-00641-z 34182989 PMC8240313

[CIT0032] Mohd, T., Al-Asiri, A., Yahya, F., Algadhi, A., Saleh, H., Saymah, A. et al., 2024, ‘Nursing around the world: Cultural variations in care’, *Power System Technology* 48(4), 3589–3602. 10.52783/pst.1212

[CIT0033] Moosavi, S., Mousavi, M.S., Ahmadi, A., Mardani, A., Parsapoor, A. & Gooshki, E.S., 2023, ‘Respecting patients’ rights in hospitals: Patients’ and health-care workers’ perspectives’, *Journal of Medical Ethics and History of Medicine* 16, 13. 10.18502/jmehm.v16i13.14308 38260764 PMC10801099

[CIT0034] O’Brien, E.M., O’Donnell, C., Murphy, J., O’Brien, B. & Markey, K., 2021, ‘Intercultural readiness of nursing students: An integrative review of evidence examining cultural competence educational interventions’, *Nurse Education in Practice* 50, 102966. 10.1016/j.nepr.2021.102966 33454512

[CIT0035] Okonji, A.M., Ishola, A.G., Ayamolowo, L.B., Femi-Akinlosotu, O.M., Mapayi, B. & Folayan, M.O., 2024, ‘Healers that hurt: A scoping review of media reports of cases of rape in healthcare settings’, *BMC Psychology* 12(1), 210. 10.1186/s40359-024-01721-w 38627793 PMC11020642

[CIT0036] Öztürk, H., Torun Kılıç, Ç., Kahriman, İ., Meral, B. & Çolak, B., 2021, ‘Assessment of nurses’ respect for patient privacy by patients and nurses: A comparative study’, *Journal of Clinical Nursing* 30(7–8), 1079–1090. 10.1111/jocn.15653 33432684

[CIT0037] Pratt, M.G., Sonenshein, S. & Feldman, M.S., 2022, ‘Moving beyond templates: A bricolage approach to conducting trustworthy qualitative research’, *Organizational Research Methods* 25(2), 211–238. 10.1177/1094428120927466

[CIT0038] Prosen, M., 2022, ‘Nursing students’ perception of gender-defined roles in nursing: A qualitative descriptive study’, *BMC Nursing* 21(1), 104. 10.1186/s12912-022-00876-4 35509039 PMC9069743

[CIT0039] Rangolo, N., Tshitangano, T.G. & Olaniyi, F.C., 2021, ‘Compliance of professional nurses at primary health care facilities to the South African cervical cancer screening guidelines’, *Nursing Reports* 11(4), 741–749. 10.3390/nursrep11040069 34968263 PMC8715466

[CIT0040] Saleem, S.G., Ali, S., Ghouri, N., Maroof, Q., Jamal, M.I., Aziz, T. et al., 2022, ‘Patient perception regarding privacy and confidentiality: A study from the emergency department of a tertiary care hospital in Karachi, Pakistan’, *Pakistan Journal of Medical Sciences* 38(2), 351. 10.12669/pjms.38.ICON-2022.5785 35310808 PMC8899898

[CIT0041] Shakwane, S., 2022, ‘Journey less travelled: Female nursing students’ experiences in providing intimate care in two nursing education institutions in Gauteng Province, South Africa’, *Health SA Gesondheid* 27(1), a1778. 10.4102/hsag.v27i0.1778 PMC8905366 35281287

[CIT0042] Sharifi, S., Valiee, S., Nouri, B. & Vatandost, S., 2021, ‘Investigating patients’ attitudes toward receiving care from an opposite-gender nurse’, *Nursing Forum* 56(2), 322–329. 10.1111/nuf.12556 33566392

[CIT0043] Soine, A., 2019, ‘The “gender problem” in nursing: Masculinity and citizenship in late Wilhelmine Germany’, *German Studies Review* 42(3), 447–467. 10.1353/gsr.2019.0076

[CIT0044] Tohit, N.F.M. & Haque, M., 2024, ‘Forbidden conversations: A comprehensive exploration of taboos in sexual and reproductive health’, *Cureus* 16(8), e66723. 10.7759/cureus.66723 39139803 PMC11319820

[CIT0045] Tomas, N., Ashipala, D.O. & Tomas, T.N., 2021, ‘Falls and fall injuries as societal challenges in Namibia’, in P. Eklund (ed.), *Integrated care and fall prevention in active and healthy aging*, pp. 242–249, IGI Global Scientific Publishing, London.

[CIT0046] Valiee, S., Moradi, Y. & Vatandost, S., 2022, ‘Female patients’ experiences of barriers to communication with male nurses: A qualitative study’, *Journal of Qualitative Research in Health Sciences* 11(4), 218–223. 10.34172/jqr.2022.09

[CIT0047] Valizadeh, F. & Ghasemi, S.F., 2020, ‘Human privacy respect from viewpoint of hospitalized patients’, *European Journal of Translational Myology* 30(1), 8456. 10.4081/ejtm.2019.8456 32499876 PMC7254454

[CIT0048] Vatandost, S., Cheraghi, F. & Oshvandi, K., 2020a, ‘Facilitators of professional communication between nurse and opposite gender patient: A content analysis’, *Maedica* 15(1), 45. 10.26574/maedica.2020.15.1.45 32419860 PMC7221273

[CIT0049] Vatandost, S., Oshvandi, K., Ahmadi, F. & Cheraghi, F., 2020b, ‘The challenges of male nurses in the care of female patients in Iran’, *International Nursing Review* 67(2), 199–207. 10.1111/inr.12582 32314370

[CIT0050] Vujanić, J., Mikšić, Š., Barać, I., Včev, A. & Lovrić, R., 2022, ‘Patients’ and nurses’ perceptions of importance of caring nurse–patient interactions: Do they differ?’, *Healthcare* 10(3), 554. 10.3390/healthcare10030554 35327032 PMC8956000

[CIT0051] Wong, M.L.L., Koh, S.L.S., Teo, W.Z., Eng, K.W. & Shorey, S., 2025, ‘Discrimination faced by male nurses and male midwives: A systematic review and meta-synthesis’, *Journal of Clinical Nursing* 34(6), 2431–2446. 10.1111/jocn.17679 39912393

[CIT0052] Xiarchi, L.M., Nässén, K., Palmer, L., Cowdell, F. & Lindberg, E., 2024, ‘Gender influences on caring, dignity and well-being in older person care: A systematic literature review and thematic synthesis’, *Nursing Philosophy* 25(1), e12467. 10.1111/nup.12467 37901941

[CIT0053] Yilmaz, S.A. & Celik, S.S., 2022, ‘Patient privacy: A qualitative study on the views and experiences of nurses and patients’, *The Australian Journal of Advanced Nursing* 39(2), 12–22. 10.37464/2020.392.447

[CIT0054] Younas, A., Ali, N., Sundus, A. & Sommer, J., 2022, ‘Approaches of male nurses for degendering nursing and becoming visible: A metasynthesis’, *Journal of Clinical Nursing* 31(5–6), 467–482. 10.1111/jocn.15958 34227187

[CIT0055] Zhang, H., Zhang, H., Zhang, Z. & Wang, Y., 2021, ‘Patient privacy and autonomy: A comparative analysis of cases of ethical dilemmas in China and the United States’, *BMC Medical Ethics* 22(1), 8. 10.1186/s12910-021-00579-6 33531011 PMC7856764

